# Resilience and quality of life in 161 living kidney donors before nephrectomy and in the aftermath of donation: a naturalistic single center study

**DOI:** 10.1186/s12882-015-0160-z

**Published:** 2015-10-16

**Authors:** Yesim Erim, Yeliz Kahraman, Frank Vitinius, Mingo Beckmann, Sylvia Kröncke, Oliver Witzke

**Affiliations:** Department of Psychosomatic and Psychotherapeutic Medicine, University Hospital, Friedrich-Alexander University Erlangen-Nürnberg, Erlangen, Germany; Department of Psychosomatic Medicine and Psychotherapy, Medical Center, University of Cologne, Cologne, Germany; Department of Psychosomatic Medicine and Psychotherapy, University Hospital Essen, University of Duisburg-Essen, Essen, Germany; Department of Medical Psychology, University Medical Center, Hamburg-Eppendorf, Hamburg, Germany; Department for Nephrology, Medical Faculty, University Duisburg-Essen, Essen, Germany

**Keywords:** Living kidney donor, Health-related quality of life, WHOQOL, Resilience, Psychosocial evaluation

## Abstract

**Background:**

Due to the shortage of cadaveric organs, living kidney donation has begun to serve as the most crucial organ pool. Transplant centers have a legitimate interest in expanding the pool of donors. A psychosocial evaluation is established in transplantation centers to prevent donors from possible emotional harm in the aftermath of donation. We explored if the resilience questionnaire is an appropriate measure of the mental stability. To standardize procedures of psychosocial evaluation and to optimize donor recruitment, we present our evaluation protocol and analyze the causes of exclusion from donation.

**Method:**

In a naturalistic design, we compared resilience and quality of life in eligible and excluded donors at the time point of donation. Potential living kidney donors (*N* = 161) participated in the obligatory psychosomatic evaluation. Quality of life (World Health Organization Quality of Life, WHOQOL-Bref) and resilience (Resilience Scale, RS-12) were measured. Three months after nephrectomy donors quality of life was screened in a follow-up.

**Results:**

In the evaluation interview donors were classified as eligible (*n* = 142) or excluded (*n* = 12). Nonrelated donors (*n* = 3) were excluded from donation significantly more often (*p* < .011). Eligible donors (M = 78.42, SD = 10.19) had higher values for resilience than excluded donors (M = 72.7, SD = 8.18, *p* < .04), who showed values comparable to the norm. In all domains of quality of life, eligible donors had significantly higher values than healthy normals (*p* < .001). After donation health-related quality of life decreased, but was comparable to the norm. A regression analysis showed that resilience was a significant predictor for all dimensions of quality of life before donation (R^2^ = 10.2–24.6 %). Post-donation quality of life was significantly correlated with pre-donation resilience scores (*p* < .05).

**Conclusions:**

The resilience score predicts high mental quality of life before and after donation. Therefor it can be implemented as a self-rating instrument to further objectify donor’s mental stability. Despite the stressful life event of donation, donor candidates presented high resilience and high levels of quality of life. Therefor our findings support health care providers` intentions to improve living donation. In the group of excluded donors nonrelated persons were overrepresented. Guidelines for the admission of nonrelated donors are currently unclear and need to be optimized.

## Background

Due to the decrease of postmortem donation and the ongoing organ shortage, living donation has begun to serve as the most crucial donor pool with increasing numbers in the western world [1, 2]. A specific advantage of living kidney donation is the superiority of its outcome compared to deceased organ donation [1]. Under these circumstances, transplant centers have a legitimate interest in expanding the pool of possible donors and first studies tailored to increase living donor transplantation have been published [3, 4]. On the other hand, the apparent medical benefits for transplant recipients have to be balanced against possible harm to living donors. A psychosocial evaluation can identify eligible donors with resilient personality traits and help to exclude psychologically vulnerable donors and finally to prevent them from possible psychological harm in the aftermath of donation [5]. There is no consensus about the scope of the psychosocial evaluation. Currently its scientific basis is insufficient and needs to be strengthened [6]. Besides a minimal standardized psychosocial screening [7] also a comprehensive evaluation of donor candidates has been recommended [8]. Therefore priority must be given to developing standards for the pre-donation psychosocial evaluation of living donor candidates.

In our previous work we researched protective factors of mental health that have been shown to facilitate healthy adjustment to life stresses as possible predictors of donors` eligibility [5, 9]. We demonstrated that resilience and social support are significant predictors of depression [9] prior to living liver donation. In the present study, resilience was surveyed as a protective factor predicting donors’ ‘quality of life’ prior to and in the aftermath of kidney transplantation.

Resilience is defined as a personality trait and a resource in coping with stress and illness according to Antonovsky`s theory of salutogenesis [10]. Rutter defined resilience as a buffering factor that protects individuals from mental distress [11]. Resilient individuals possess self-esteem, believe in one`s self-efficacy, have a repertoire of problem-solving, and satisfying interpersonal relationships. Therefore, we expected the construct of resilience to fit with the personality requirements directed to donors.

‘Quality of life’ is an assessment of how the individual's well-being may be impaired by a disease or disability and makes a comparison of the reported actual life situation with the highest possible perceived well-being over the lifespan. Recent comprehensive reviews certify that kidney donors are of good mental health, without occurrence of depressive or anxiety symptoms in the majority. But these reports include also unfavorable psychological results such as depressive reactions and fatigue in a small proportion of donors [12–14]. Altogether protective and situative factors fostering positive psychosocial outcome are not sufficiently surveyed.

The objective of the present study was to find out if the resilience questionnaire is an appropriate measure of the mental stability and hardiness of the donors. Therefore we compared eligible and excluded kidney donors in relation to resilience and quality of life. A representative German adult cohort served as the control group for each questionnaire. We assumed that donor candidates would express higher levels of resilience and health-related quality of life than the norm population, and that eligible donors would score higher on these parameters than the rejected donors. Furthermore a follow-up screening was made to analyze associations of pre-donation resilience with post-donation quality of life outcomes. A second objective was to standardize donor evaluation procedures and to optimize donor recruitment. Therefore, we present our evaluation protocol and analyze the causes of exclusion from donation.

## Methods

### Study design and sample

We examined from July 2009 to December 2012 161 potential living kidney donors who were admitted for a psychosomatic screening to the Department of Psychosomatic Medicine and Psychotherapy in Essen. During the psychosomatic interview, which is a substantial part of the preoperative evaluation of donors, they were asked to take part in the study and gave their written informed consent. Two sources of information of donor evaluation for eligibility were included in the study. First, there are the results of a comprehensive psychosomatic interview with a judgment for either acceptance as a living kidney donor or exclusion. Second, we used patient reported outcomes, ascertained with questionnaires on quality of life and resilience. Three months post-donation donors’ quality of life was screened again. The local Ethical Committee of the University Hospital of Essen (approval number:02–2030) approved the study protocol. Inclusion criteria were a good command of the German language and a permanent residence status in Germany. All eligible donors agreed to study participation and were included. Seven donors were excluded on the basis of missing values (30 % of the questionnaires items). In our study only eligibility due to psychosocial issues has been considered. Exclusions because of physical impediments have not been taken into account.

### Donor evaluation procedures

Our study group established a standardized procedure (Table 1) for the clinical evaluation interviews [5]. The psychosomatic interviews are carried out at step 2 and 4. In step 2 donor and recipient are evaluated separately by two different interviewers. This procedure allows to focus on the respective perspective of the donor or the recipient and on the relationship between them. The interview includes life situation, biographical and psychiatric history and the examination of the mental stability of the donor. It is clarified if the donor has been sufficiently informed on the surgical procedure and on possible complications. If the donor proceeds in the medical examinations, in step 4, the two evaluators meet together with the donor-recipient-couple and examine the dynamics of their relationship and their expectations concerning the transplantation. After this procedure has been concluded the donor is presented to an external ethical board before the medical association. The so called transplantation ethics committee examines possible pressure to donate in the family and organ trading.

**Table 1:** Evaluation protocol for potential living kidney donors - Course of the psychosomatic assessment

		Content	Carried out by
Step 1	First information (informed consent)		Nephrologist/ Surgeon
Step 2	First psychosomatic assessment	1. Mental stability, actual psychosocial situation	Different evaluators (two psychosomatic specialists) for donor and recipient
		2. Scrutinization and verification of the informed consent	
		3. Verification of voluntariness taking relative behavioral patterns and neuroticisms of the donor into consideration	
Step 3	Other medical examinations		
Step 4	Second psychosomatic assessment	1. Dynamics of the relationship between donor and recipient	Donor, recipient and their evaluators (=advocates)
		2. Anticipation of the transplantation	
Step 5	Transplantation board	External assessment consisting of	Members of the transplantation board by the general medical association (a physician, a magistrate, a psychologist or specialist of psychosomatics)
	Evaluation by the general medical association	- verification of voluntariness	
		- exclusion of financial interests	
Final Informed consent	Last preparations prior to the operation		Anesthesiologist, Surgeon

### Study measures

#### Resilience scale, German version (RS-13)

The RS-13 is a short version of the Resilience Scale (RS-25) [15] and measures the competence to moderate the negative effects of stress, and acceptance of life and self. The German version has very good internal consistency (Cronbach’s α = .90) [16].

#### World health organization quality of life, German version (WHOQOL-bref)

For evaluating quality of life we used the German version of WHOQOL-Bref [17, 18], which includes four domains such as physical health, psychological health, social relationships and environmental conditions. The items are rated on a five point Likert scale, with a high score indicating better quality of life. Cronbach’s alpha of the German version is between α = .57 and .88 [18].

### Statistical analysis

All scoring and statistical analyses were performed using the Statistical Package of Social Sciences 21 (SPSS Inc., Chicago, IL, USA). The data for descriptive analyses were shown as mean values, standard deviations and percentage values. On the basis of the unequal sample sizes of the two groups, for group comparisons we used the Mann–Whitney U-test for nonparametric sample size. For comparisons with norm values and between the groups we used the one-sample t-test or the t-test for independent samples, respectively. Chi-square tests were applied for categorical variables. We also calculated Cohen´s d for estimating the effect size. Analyses of the relationship between the dependent and independent variables were performed with Pearson and Spearman correlations. To detect the predictors for quality of life we used linear regression analyses to quantify the contribution of resilience and sociodemographic features. A significance level of 0.05 was predetermined. For alpha adjustment we used the Bonferroni-Holm correction [19].

## Results

### Demographic features

The total sample, after removal of incomplete questionnaires, consisted of 154 potential donor candidates. Sociodemographic characteristics of the sample are depicted in Table 2. After the evaluation interview, donor candidates were classified as either eligible (*n* = 142) or excluded (*n* = 12).

**Table 2:** Demographical features

	Donor candidates (*n* = 154)	Eligible donors (n =142)	Excluded donors (*n* = 12)	Statistical comparison
Age				
mean (SD)	50.9 (10.09)	50.72 (10.34)	53.42 (6.34)	n.s.
range	23 – 72	23 – 72	43 – 61	
Gender				
female	86 (55.8 %)	78 (54.9 %)	8 (66.7 %)	n.s.
male	68 (44.2 %)	64 (45.1 %)	4 (33.3 %)	
Family status				
single	11 (7.1 %)	10 (7.0 %)	1 (8.3 %)	n.s.
married	115 (74.1 %)	107 (75.4 %)	8 (66.7 %)	
widowed	4 (2.6 %)	4 (2.8 %)	-	
separated/ divorced	24 (15.6 %)	21 (14.8 %)	3 (25 %)	
no data	-	-	-	
Education level				
without certificate	4 (2.6 %)	4 (2.8 %)	-	n.s.
middle school	108 (70.6 %)	96 (67.7 %)	12 (100 %)	
university-entrance	41 (26.8 %)	40 (28.2 %)	-	
diploma				
not reported	1 (0.6 %)	2 (1.4 %)	-	
Nationality				
German	145	133 (93.7 %)	12 (100 %)	n.s.
others	9	9 (6.3 %)	-	
Donotation for				
parents for children	40 (26.5 %)	38 (26.8 %)	2 (16.7 %)	.049*
children for parents	1 (0.7 %)	1 (0.7 %)	-	
spouses	59 (38.3 %)	57 (40.1 %)	2 (16.7 %)	
partner	9 (5.8 %)	8 (5.6 %)	1 (8.3 %)	
siblings	27 (17.9 %)	24 (16.9 %)	3 (25 %)	
other relatives	8 (5.3 %)	7 (4.9 %)	1 (8.3 %)	
non-relatives	7 (4.6 %)	4 (2.8 %)	3 (25 %)	
no data	3 (1.9 %)	3 (2.1 %)	-	
Recipients				
on dialysis	110 (71.4 %)	99 (69.7 %)	11 (91.7 %)	
pre-emptive	40 (26 %)	40 (28.2 %)	-	
no data	4 (2.6 %)	3 (2.1 %)	1 (8.3 %)	

* Fischer`s z-value

### Reasons for the exclusion of donors

#### Lacking mental stability

Seven donor candidates were rejected for mental vulnerability. Four of them presented different psychiatric diagnosis of previous or ongoing disease. One candidate in this group wanted to have a medical excuse to decline donation without offending his help seeking relative. Two other candidates were assessed as being vulnerable and not stable enough although they did not show previous psychiatric diagnosis or treatment but presented with unrealistic expectations.

#### Continuous ambivalence

Two donor candidates were excluded because of ambivalence; in the confidential psychosomatic interview they reported not being able to make a decision. If this indecisiveness could not be explained or reduced after two or more counseling sessions of 50 min each with the psychosomatic interviewer, it was suggested that they decline donation as a means of mental relief.

#### Personal closeness not substantiated

Three non-related candidates were excluded from donation because their personal closeness to the recipient could not be substantiated. Due to the German transplantation law, living organ donation is allowed only for family members or a person who have a so called “obvious close relationship” that means a significant long term relationship to the recipient. These three donor candidates, however, had given the impression that they had a coincidental relationship to the recipient. One of them was also regarded as being mentally vulnerable. Nonrelated donors were excluded from donation significantly more often (*p* < .011).

Psychiatric diagnoses according to ICD-10 [20] and reasons of exclusion are summarized in Table 3.

**Table 3:** Excluded kidney donors (*n* = 12)

Age (yrs)	Gender	Donation for	ICD-10 Diagnoses	Reason for exclusion
44	♀	Partner	Substance dependency F19.20	Lacking mental stability
43	♀	Husband		Lacking mental stability
59	♀	Child		Conflict of ambivalence
56	♀	Husband		Conflict of ambivalence
55	♀	Friend	Recurrent depressive disorder F33.1 Somatoform pain disorder F45.40	Lacking mental stability
48	♂	Non-related		No obvious close relationship
54	♀	Child		Lacking mental stability
61	♀	Non-related		No obvious close relationship Lacking mental stability
61	♀	Non-related		No obvious close relationship
56	♂	Sibling	Recurrent depressive disorder F33.1	Lacking mental stability
57	♂	Sibling	Alcohol dependency F10.20	Lacking mental stability
47	♂	Child	Alcohol dependency F10.20	Lacking mental stability
				Medical excuse

#### Resilience

**Fig. 1:**
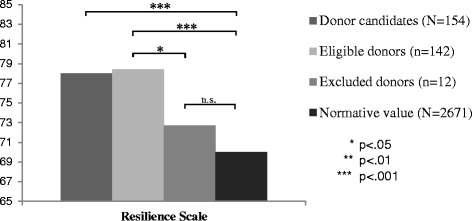
Mean values of the Resilience Scale in potential living kidney donors: Eligible vs. excluded donors

Considering the whole group, the donor candidates (M = 78.04, SD = 10.18) had mean values for resilience significantly higher than norm values (M = 70.0 SD = 12.0, *p* < .001). Compared to values of the normative population, the mean score of resilience for eligible donors M = 78.42, SD = 10.19) was significantly higher (*p* < .001). Resilience scores of the excluded donors (M = 72.7, SD = 8.18) were comparable to the norm. Eligible and excluded donors did also differ significantly from each other (*p* < .04). Results are depicted in Fig. 1 and the gender-specific comparison is presented in Table 4.

**Table 4:** Gender-specific mean scores of resilience and quality of life in comparison to the norm

	Female donor candidates (A)		Female German population (B)		Male donor candidates (C)		Male German population (D)		Group comparison
	Mean	(SD)	Mean	(SD)	Mean	(SD)	Mean	(SD)	
Resilience scale	79.54	(9.21)	-^b^		75.69	(11.09)	-^b^		A > C***
Physical QoL^a^	85.49	(11.48)	75.35	(18.13)	86.36	(11.02)	78.84	(16.93)	A > B***; C > D ***; A = C
Psychological QoL^a^	78.71	(12.84)	72.49	(16.28)	81.67	(11.44)	75.88	(14.72)	A > B***; C > D ***; A = C
Social QoL^a^	79.75	(15.04)	71.41	(18.78)	79.59	(14.38)	72.34	(18.21)	A > B***; C > D***; A = C
Enviromental QoL^a^	83.63	(12.38)	69.73	(14.05)	82.15	(11.28)	71.17	(14.28)	A > B***; C > D ***; A = C

a: Domains of WHOQOL-Bref; b: gender-specific values are not available; *p < .05; **p < .01;***p < .001

#### Quality of life

In comparison with values of the norm population for quality of life (physical health: M = 76.92, SD = 17.68; psychological health: M = 74.02, SD = 15.68; social relationships: M = 71.83, SD = 18.52; environmental: M = 70.38, SD = 14.17), the mean values for the whole group indicated a significantly better quality of life, with a large effect size for environmental conditions (M = 82.97, SD = 11.09; *p* < .001, d = .88), a middle effect size for physical health (M = 85.87, SD = 11.25; *p* < .001, d = .52), a small to medium effect size for social relationships (M = 79.69, SD = 14.70; *p* < .001, d = .43) and psychological health (M = 80.01, SD = 12.29; *p* < .001, d = .39).

**Fig. 2:**
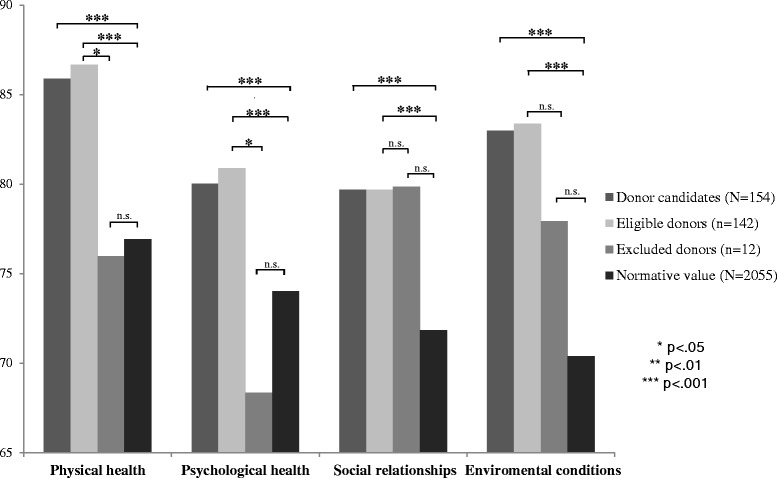
Mean values of Quality of life in potential living kidney donors: Eligible vs. excluded donors

In all domains of quality of life, eligible donors had significantly higher values than the normative sample (physical health: M = 86.66, SD = 10.29; *p* < .001; psychological health: M = 80.89, SD = 11.71; *p* < .001; social relationships: M = 79.67, SD = 14.68, *p* < .001; environmental: M = 83.37, SD = 11.62; *p* < .001). The domain scores of the excluded donors (physical health: M = 75.97, SD = 17.57; psychological health: M = 68.94, SD = 14.72; social relationships: M = 79.86, SD = 15.67; environmental conditions: M = 77.92, SD = 14.54) were comparable to norm values. The group comparisons for physical (p = .034, d = .98) and psychological health (*p* = .005, d = .99) demonstrated a higher quality of life with a larger effect size for eligible donors than for excluded donors. Comparable values were achieved in both groups only for the subscales environmental conditions and social relationships. Results are depicted in Fig. 2. The alpha adjustment for multiple testing according to the Bonferoni-Holm-correction did not change the significance of results. Gender-specific mean scores of pre-donation quality of life are presented in Table 4.

Concerning mental quality of health donors for recipients on dialysis (*n* = 110; M = 81.61, SD = 11.30) or for preemptive transplantation (*n* = 40; M = 78.44, SD = 12.62) didn’t differ from each other (*p* < .151).

**Fig. 3:**
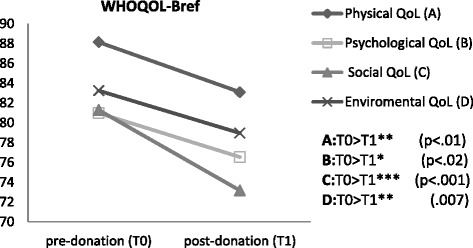
Health-relatd Quality of life: Progression after donation

Three months after donation health-related quality of life was significantly impaired in all domains compared to pre-donation values (physical health: M = 83.1, SD = 12.5; psychological health: M = 76.54., SD = 14.87; social relationships: M = 73.17, SD = 17.13; environmental conditions: M = 78.96, SD = 11.16), but still within the standard norm values. Results are depicted in Fig. 3.

#### Correlational analysis

Age was correlated negatively to higher scores of physical health. Male gender was positively correlated with resilience. Scores of resilience were positively correlated to all subscales of quality of life. The correlation matrix is depicted in Table 5.

**Table 5:** Correlations among health-related quality of life, resilience and sociodemographic features for the total sample (*N* = 154)

	Resilience	Age	Gender	Physical QoL^a^	Psychological QoL^a^	Social QoL^a^
Age	.064					
Gender	.201*					
Physical QoL^a^	.420***	-.216*	-.039			
Psychological QoL^a^	.469***	-.096	-.120	.703***		
Social relationships QoL^a^	.329**	-.069	.005	.501***	.625***	
Environmental QoL^a^	.421***	-.047	.062	.527***	.612***	.599***

^a^Domains of WHOQOL-Bref * < .05 level (two-tailed) ** < .01 level (two-tailed) *** < .001 (two-tailed)

#### Post-nephrectomy follow-up

Out of the whole group of potential kidney donors (*n* = 161) 111 have undergone a nephrectomy. 41 (46.7 %) donors responded to follow-up questionnaires by mail. Three months after donation, all domains of health related quality of life were correlated significantly with pre-donation resilience score (psychological health = .380, *p* < .05; social relationships = .386, *p* < .05; environmental conditions = .325. *p* < .05), except for physical quality of life.

#### Regression analyses

For exploring the association between pre-donation quality of life as well as resilience, gender, and age, a stepwise regression model was applied. Results of the stepwise regression analyses are given in Table 6.

**Table 6:** Stepwise multiple regression analyses for the total sample (*N* = 154)

Dependent variable	Significant predictors	beta	p	Adj. R^2^
Model 1^b^	1. Resilience	.428	.001	21.8 %
Physical Health^a^	2. Age	-.230	.001	
Model 2^b^	1. Resilience	.502	.001	24.6 %
Psychological Health^a^	2. Gender	-.195	.001	
Model 3^b^	1. Resilience	.329	.001	10.2 %
Social relationships^a^				
Model 4^b^	1. Resilience	.421	.001	17.2 %
Environmental conditions^a^				

^a^Domains of WHOQOL-Bref. ^b^Independent variables in each regression model: Resilience, Age, Gender

The first regression analysis explained 21.8 % of the variance regarding physical health with resilience (ß = .428; *p* < .001), and age (ß = −.230; *p* < .001) as significant predictors.

In the second regression analysis for psychological health 24.6 % of the variance was explained, with resilience (ß = .502; *p* < .001), and male gender (ß = −.195; *p* < .001) as significant predictors.

The regression analysis for social relationships explained 10.2 % of the variance with resilience (ß = .329; *p* < .001) as a significant predictor.

Resilience (ß = .421; *p* < .001) was also a significant predictor for environmental conditions of quality of health with an explained variance of 17.2 %.

## Discussion

To our knowledge, this is the first study of living kidney donors to present results of the clinical psychosocial evaluation together with patient reported outcomes. A special objective was to characterize the differences between the groups of the eligible and excluded donor candidates and outline the sample of the rejected patients.

The primary strength of our study is a large sample with a 100 % response rate, with all donor candidates who fulfilled the inclusion criteria consenting to study participation. This high acceptance rate may be due to socially desired behavior in order to pass the donor evaluation prior to transplantation. On the other side, it shows the acceptance of the donors towards the psychosomatician as an advocate and interlocutor during the screening procedures. The whole sample consisted of 55.8 % women and therefore did not present a significant gender disparity. Some previous research has reported higher rates of female donors [21]. In times when women contributed less to the family income, they might have been considered as an organ donor more often than the male members of the family. The proportions of gender may be equalizing due to changing perceptions of gender roles in society.

The donor selection process has been pointed out as an important limitation factor of living donor transplantation [22]. In our survey, previous mental illness or ongoing signs of mental instability were the most frequent causes of exclusion, together with nonrelated donors whose personal relationship to the donor could not be ascertained.

Standardized procedures for the psychological evaluation of living kidney donors do not exist and reports about evaluation of donors’ eligibility in the literature are rare. It is therefore not surprising that exclusion rates for psychosocial reasons are varied. In a transplantation program in London [23], voluntary withdrawal of 42 (27 %) of the prospective donors was the commonest reason for non-donation. In a US center analysis of donor evaluation [24] 47 % of prospective donors were excluded, 22 (5 %) of these on the basis of psychosocial reasons. In our study only psychosocial eligibility was focused and 12 (8 %) of donors were excluded. This is comparable to the US center results. The respectively low exclusion rate can putatively be explained by the first nephrology information session which has the function of a pre-screening. Especially in cases when recipients are on dialysis, treating nephrologists are well informed about family and environmental conditions.

Concerning the socio-demographic characteristics of the donors, a relevant group in our survey was the nonrelated donors. Three nonrelated candidates were excluded on the basis that their personal closeness to the recipient was not substantiated; one was also deemed not to have sufficient mental stability. In contrast to the USA, where living donation is not limited to donor-recipient pairs with long-standing emotional relationships, the German transplantation law stipulates an “obvious individual relationship” between the recipient and the donor. Donors without a prolonged emotional relationship to the recipient are therefore excluded from donation. Reasons for this procedure are concerns about covertly accepted financial profit or secret coercion, which are difficult to clarify in unrelated donation.

Eligible and excluded donors presented high scores on resilience, with eligible donors even exceeding the normative values. This result is in agreement with previous research where donors had high levels of mental health-related quality of life, prior to and in the aftermath of donation [12]. In a recent investigation by our study group, living liver donors demonstrated values of resilience comparable to the norm, and low levels of mental distress, measured as depression or anxiety [9]. Rudow et al. [25] established resilience levels similar to the general population in a mixed population of living liver and kidney donors, after donation, however the study results were limited due to poor response rates, which in our study could be overcome.

In analyzing the different domains of quality of life, high effect sizes in comparison to the norm were maintained in environmental factors, which include general living conditions. In many stages of organ donation, e.g. in the post-transplant period, families need good emotional and instrumental support and resources. Putatively good living conditions and resources are a necessary precondition for families to consider living organ donation. For the domain ‘physical health’, a medium effect size was observed. Small to medium effect sizes were established for the domains ‘social relationships’ and ‘psychological health’. These results show that differences in self-reported outcomes between the groups are small. However, eligible donors achieved significantly higher scores for physical and psychological quality of life than excluded donors, an outcome that strongly supports the soundness of our clinical evaluation interview.

Male gender predicted higher psychological quality of life. This is in line with the results of a recent follow-up survey 8 to 9 years after transplantation. The authors reported emotional summary score for quality of life was lower in female donors, caused by a reduced role functioning [26]. The world-wide higher incidence of depressive disorders in women may explain the differences [27, 28]. Women may be burdened by multiple familial role requirements in the context of donation, e.g. as donors and simultaneously as care giving marital partners. Nevertheless this finding requires further investigation and women should be regarded as a risk group.

Looking at the whole sample pre-donation health-related quality of life was higher than in the normative sample. This finding is in line with a recent study showing that kidney donors present high levels of emotional and physical functioning before transplantation [29].

Only 46.7 % of donors responded our follow-up questionnaires. Incomplete follow-up information for donors has been recognized in many centers and in the United States one year post-donation information was eligible for only 66.8 % for living kidney donors in the period from 2008 to 2009 [30]. Lacking motivation of donors who prefer to be treated by their own local physician rather than the transplant program was suggested as an explanation. In our center some donors reported they wanted to cope with the donation experience by themselves.

Three months after nephrectomy donors showed a significant decline in quality of life. This may be due to the early time point of our measurement. Similar results were shown by Lumsdaine et al. [31] who proved quality of life of kidney donors reduces to UK normative levels 6 weeks after operation. In that cohort the scores improved again at 1 year. Other authors reported that, only a small proportion of kidney donors had adverse outcomes in psychosocial health after transplantation [12]. A large-scale multi-center study [13] established kidney donors’ quality of life outcomes to be equal to or exceeding normative values, with the mental component staying stable over time. In recent studies mental health outcome of donors have been compared to a matched population of healthy individuals. Also with this method outlined changes in mental health of donors after transplantation did not differ from the fluctuations found in the general population [7]. Furthermore, the physical quality of life of the donors remained stable [32]. Compared with healthy non-donors kidney donors had an increased risk of end stage renal disease, but the magnitude of the absolute risk increase was small. It must be noted, that post-nephrectomy quality of life was measured between 1 and 48 years after transplantation in those studies [13, 29, 32]. Our results indicate donors may have higher distress levels in the early period after nephrectomy. Psychosocial support may be most necessary at this point in time. On the other hand donors report high quality of life comparable to the norm even in this moment. This excellent quality of life outcome after transplantation can be explained by the perfect health satisfaction prior to transplantation as measured in our survey.

In accordance with our expectations, resilience was significantly correlated with all dimensions of pre-donation health-related quality of life. The higher the resilience, the higher the domain scores of health-related quality of life. Regression analyses revealed resilience as a significant predictor of all domains of pre-donation health-related quality of life. These findings are in line with its psychological construction as a buffering or mediating factor between actual burden and the degree of distress symptoms expressed [33]. Even though organ donation is a stressful life event, candidates possessing high resilience perceived themselves as having a high level of quality of life at the time-point of donor evaluation. For a perfect comparability of such reports the psychological assessment of donor candidates should be conducted in standardized steps as we suggest with our assessment procedure.

One important limitation of our study is that only 46.7 % of the donors responded to the post- donation screening. Secondly the tendency to report toward socially accepted behavior may lead to an understatement concerning mental distress and to exaggeration of personal strengths in donors. Furthermore, this was a single center study and the group of excluded donors could be too small to detect significant results. In future, efforts should be made to establish standardized evaluation criteria and procedures that would enable researchers to compare outcomes of donors from different transplant centers and countries.

## Conclusions

In our survey only 8 % of the donor candidates were excluded and the majority of the group showed high levels of resilience and quality of life, therefor we affirm that kidney donor candidates constitute a resilient population of good mental health. The correlation of pre-donation resilience with post-donation quality of life hints at the predictive value on resilience, but this finding has to be verified in a sample with higher post-donation study response.

We employed a quality of life questionnaire to measure life satisfaction and psychological well-being and further a resilience questionnaire as a protective factor of mental health. Our results show that the resilience scale can be implemented as a self-rating instrument in the psychosomatic evaluation of donors and would help to further objectify donors` mental stability. Nevertheless, clinical evaluation should remain the central instrument of evaluation in the organ donation setting. Psychometric questionnaires cannot replace communication with the donors. Finally, with regard to the legitimate admission of nonrelated donors our study shows the urgent necessity for nationwide and international standardization of donor evaluation.

## Abbreviations

ICD-10: International Statistical Classification of Diseases and Related Health Problems, tenth revision
RS: Resilience Scale
SPSS: Statistical Package of Social Sciences
WHOQOL-Bref: World Health Organization Quality of Life, short version
